# The Therapeutic Landscape in Chronic Cough

**DOI:** 10.1007/s00408-023-00666-y

**Published:** 2023-12-21

**Authors:** Jaclyn A. Smith

**Affiliations:** 1 Division of Immunology, Immunity to Infection and Respiratory Medicine, University of Manchester and Manchester Academic Health Science Centre, Wythenshawe Hospital, Southmoor Road, Manchester, M23 9LT UK; 2 Manchester University NHS Foundation Trust, Manchester, UK

**Keywords:** Antitussives, Refractory chronic cough, Idiopathic pulmonary fibrosis, P2X3 antagonists

## Abstract

In recent years, there has been a substantial increase in the development of antitussive therapies and the first new therapy, gefapixant has been licenced in Europe. This review describes current unlicenced treatments for chronic cough and details treatments currently in development for refractory chronic cough and cough in idiopathic pulmonary fibrosis, as well as compounds previously explored.

## Introduction

### Background

Chronic coughing is a common complaint, affecting approximately 4–10% of the general population and associated with factors, such as smoking, respiratory diseases, low income, and occupational exposures [1, 2]. It is thought that about 5% of those with chronic cough have chronic coughing that is either unexplained or refractory to treatment of co-morbid conditions, the most common of which are asthma, chronic rhinosinusitis, and gastro-oesophageal reflux disease. It is these patients who have a need for antitussive therapies to control their coughing. For the purposes of this manuscript the term refractory chronic cough (RCC) will be used to describe such patients and includes those in whom no underlying condition can be identified. The first therapy ever to be developed and licenced for the treatment of RCC, gefapixant, has recently been licenced in the European Union in addition to Switzerland and Japan. At the time of writing, an FDA advisory committee review of this therapy is imminent. As a result of these developments, the interest in new cough treatments has rapidly expanded. However, the availability of these treatments for patients, after licencing, will still depend upon the payers and health economic assessments.

### Who Needs Cough Treatments?

**Fig. 1:**
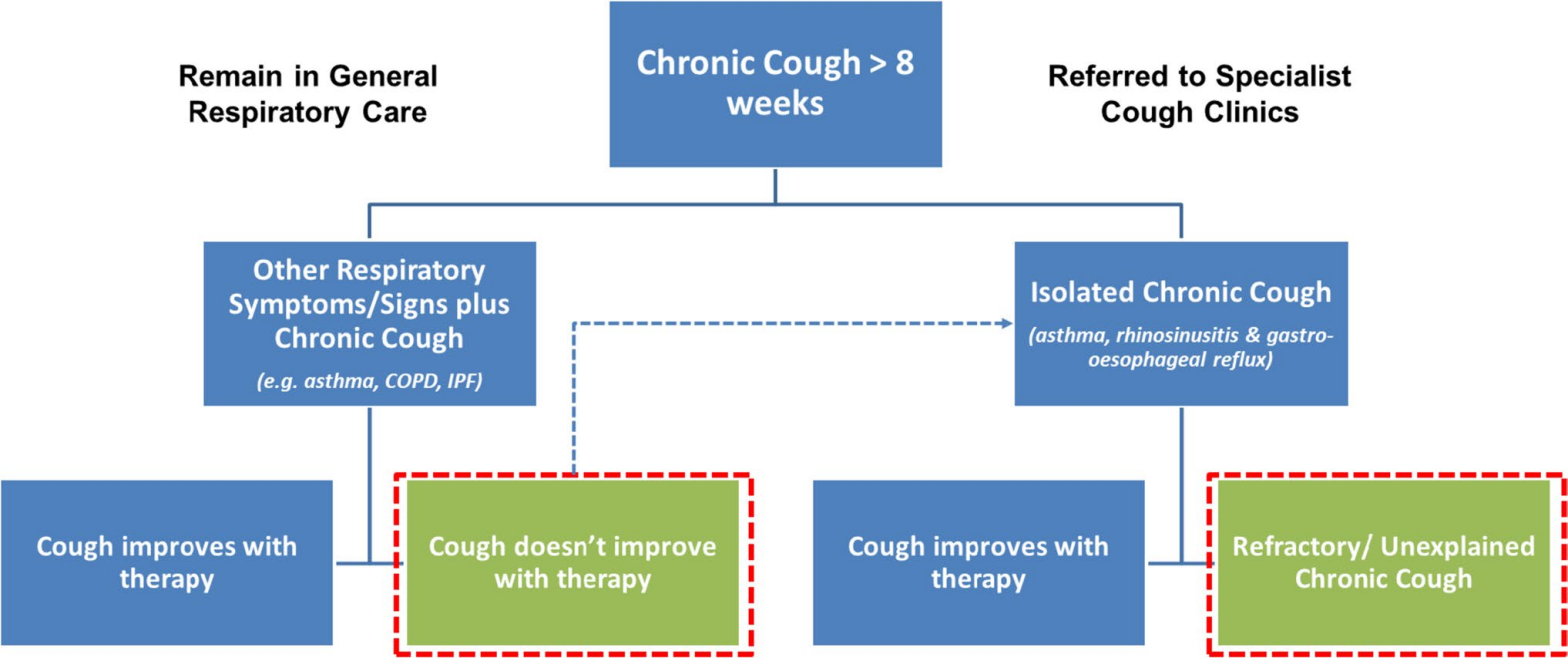
Clinical pathways of patients requiring cough treatments; *COPD* chronic obstructive pulmonary disease, *IPF* idiopathic pulmonary fibrosis

The development of therapies for chronic cough has, understandably, focussed on patients presenting with chronic cough as their main complaint and following evaluation and treatment trials are found to have RCC [3]. In some countries, these patients are managed in specialist cough clinics. Chronic coughing is also fairly ubiquitous in respiratory disease beyond the commonly associated co-morbidities of asthma, chronic rhinosinusitis, and gastro-oesophageal reflux disease. Patients diagnosed with common respiratory diseases may also experience significant coughing that is not addressed by standard care for their condition and can be sufficiently troublesome to warrant specific treatment, Fig. 1. Few of these patients have access to specialist cough clinics and therefore may have poorer access to specific antitussive interventions. Perhaps the best example of such a condition is idiopathic pulmonary fibrosis where a significant subgroup of patients suffer chronic coughing alongside progressive breathlessness [4]. Cough frequencies in IPF have been reported to be comparable to those seen in patients presenting to chronic cough clinics and there is little evidence to suggest antifibrotic therapies improve this symptom [5, 6]. Similarly, in some patients with airways disease excessive coughing is also troublesome and not always responsive to standard care [7–9]. This may result in inappropriate escalation of treatment with no real benefit. It is increasingly accepted that cough hypersensitivity (defined as coughing to relatively innocuous stimuli) may represent a treatable trait in asthma and COPD patients [10]. The precise mechanisms driving cough in patients with RCC, IPF, and airways disease are likely to be different from one another and may require different therapeutic interventions, but this needs further exploration [11].

### Mechanisms of Action of Antitussive Therapies

#### Peripheral Nervous System Targets

Most of our understanding of the neurophysiology of cough has been derived from studies in guinea pigs which have identified the relevant neurotransmitters and receptors to be targeted for antitussive therapies [12]. Coughing is mediated by sensory vagal fibres innervating the larynx and proximal airways that can be broadly divided into chemically sensitive C fibres and mechanically sensitive Aδ fibres. C fibres are characteristically activated by capsaicin, the pungent extract of chili peppers, via the transient receptor potential vanilloid (TRPV1) ion channel; they are also responsive to changes in temperature and pH via this receptor. C fibres express a wide range of other ion channels and G-protein-coupled receptors, including transient receptor potential ankyrin 1 (TRPA1) which is activated by chemical irritants such as acrolein, cinnamaldehyde, mustard oil, and constituents of perfumes, cleaning products, and diesel fumes which are commonly reported to evoke coughing in chronic cough patients. They are also responsive to endogenous inflammatory mediators such as prostaglandins and bradykinin through EP3 and B2 receptors and to adenosine triphosphate (ATP), an alarmin released by cell stress and injury, through P2X3 channels.

In contrast, Aδ fibres are predominantly responsive to mechanical stimuli, such as airway mucus and inhaled foreign bodies, although the mechanism of mechanical transduction is unclear. In addition, they respond to low pH through acid sensing ion channels (ASICs). In both Aδ and C fibres, the activation of ion channels and receptors leads to generator potentials which through the opening of voltage-gated sodium channels are converted to action potentials. Voltage-gated sodium channels are also responsible for the conduction of action potentials along the axons to the central nervous system.

In patients with RCC, there is some evidence of a role for peripheral neuronal sensitisation. For example, one study as shown increased airway nerve branching and density in endobronchial biopsies from RCC compared with healthy controls [13]. The airways are therefore hyper-innervated. Furthermore, the effectiveness of P2X3 antagonists, which are thought to be poorly penetrant in the central nervous system, implicates peripheral P2X3 receptors and ATP in the airway.

**Fig. 2:**
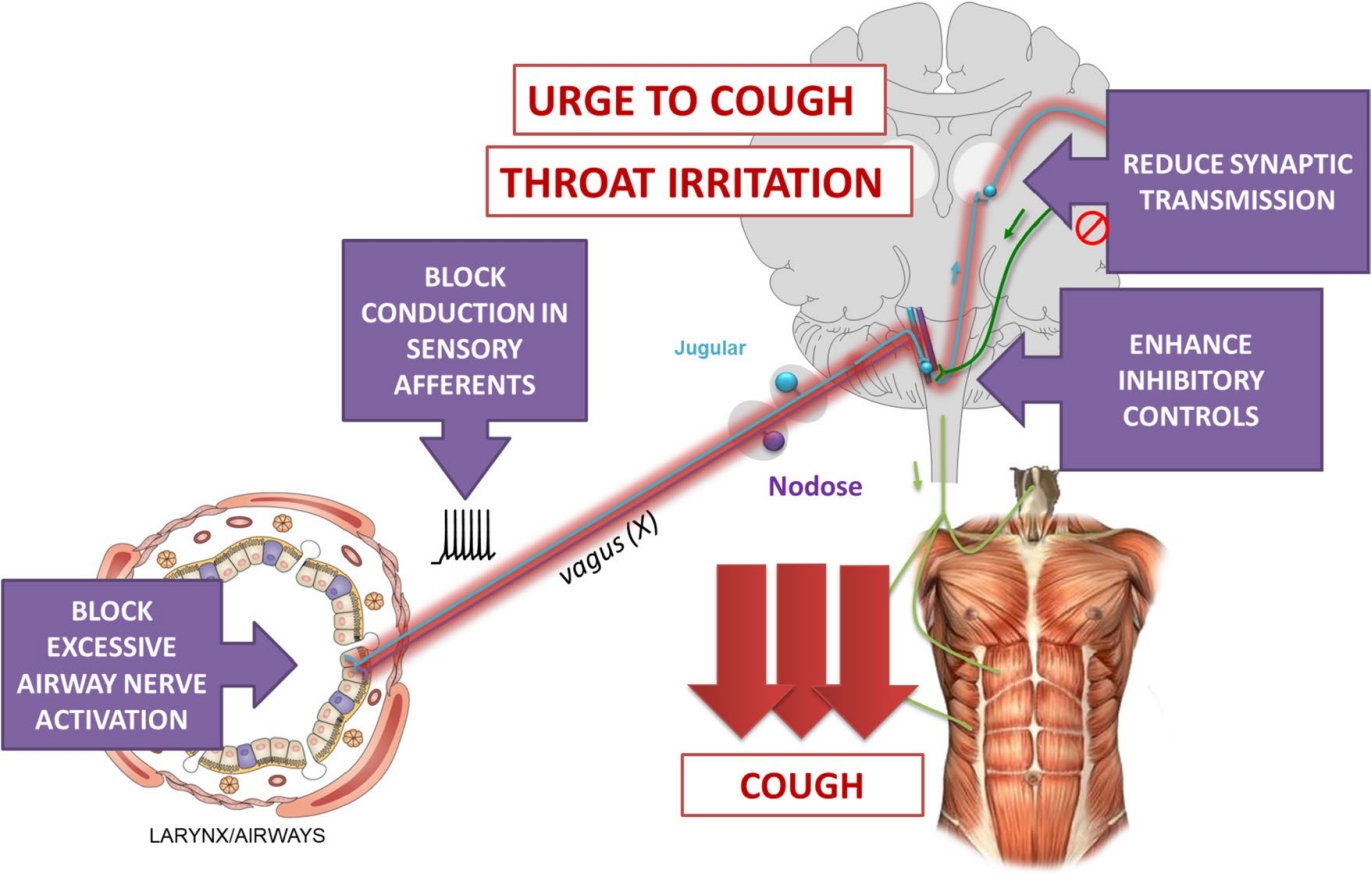
Mechanisms of action of antitussive therapies

Most of the antitussive therapies developed in recent years have targeted receptors on peripheral nerves, blocking their activation by endogenous and exogenous ligands in the airways or reducing conduction of action potentials, see Fig. 2. This approach reduces the adverse effects often associated with therapies active in the central nervous system, but establishing which of the many cell surface receptors expressed by airway nerves are most important in driving chronic coughing is difficult.

#### Central Nervous System Targets

Afferent vagal fibres from the airways synapse in the brainstem in the nucleus tractus solitarious and paratrigeminal nucleus. In the brainstem, glutamate and substance P are thought to be released by the proximal terminals of these fibres, activating post-synaptic neurones through AMPA (α-amino-3-hydroxy-5-methyl-4-isoxazolepropionic acid), NMDA (*N*-methyl-d-aspartate), and neurokinin (NK) receptors. Brainstem nuclei are responsible for coordinating the production of the cough motor pattern, whereas higher centres mediate the sensations associated with airway irritation, the urge-to-cough, the behavioural regulation of cough, and associated cognitive and affective consequences. In humans, the inhalation of cough-evoking stimuli such as capsaicin produce widely distributed brain activity in functional brain imaging studies, including the primary and secondary sensory cortex and cingulate, insula, and orbitofrontal cortices [14]. There is some evidence of amplified central nervous system responses to inhaled capsaicin in patients with chronic cough compared with healthy volunteers [15]. Moreover, RCC patients seem to be indiscriminately hyperresponsive to a wide range of tussive challenges and report triggering of cough with many different environmental irritants [11, 16]. This is most in keeping with sensitisation of central mechanisms.

Cough can be consciously suppressed but is also subject to unconscious inhibitory control mechanisms, well described in other fields including those involved in placebo effects and in response to pain (conditioned pain modification). There is evidence that RCC patients have an impaired ability to consciously supress coughing [17, 18] and but also reduced cough inhibition to painful stimuli [19]. Based on work in other fields such as chronic pain, opioid, and gamma amino butyric acid type B (GABA_B_) receptors may be implicated in these inhibitory pathways.

Centrally acting drugs to treat cough may act to reduce amplification of action potentials at synapses in the central nervous system and or activate deficient inhibitory control mechanisms, see Fig. 2.

### Is it Safe to Suppress Coughing?

There is a risk in patients with productive cough, for example, chronic bronchitis and bronchiectasis and those within impaired swallow, that reducing coughing could impair the clearance of airway secretions and airway protection, leading to lower respiratory tract infections. This risk is probably greatest with treatments that broadly suppress coughing, irrespective of the precise peripheral mechanisms provoking cough; centrally acting treatments and lidocaine-like therapies being most likely to raise these concerns. As a result, trials of novel antitussive therapies have only recruited those with dry or minimally productive chronic cough and exclude conditions characterised by mucus hypersecretion or associated with neurological conditions. Drugs with a specific peripheral mode of action that reduces the hyper-excitability of afferent pathways are unlikely to cause these issues, and indeed in studies to date there has been no signal suggesting increased respiratory infections in treated patients. Furthermore, even the most effective therapies do not return cough frequency to that seen in healthy volunteers which is typically only about five coughs per day [20].

### How are Cough Treatments are Evaluated?

The study designs and endpoints utilised to evaluate therapies for chronic cough have evolved substantially in the last 10 to 15 years. To describe these in detail is beyond the scope of this review. In brief, regulatory bodies require a thoroughly validated objective cough monitoring system using a CE marked/FDA 510 K registered device as the primary endpoint in regulatory studies to demonstrate a therapy reduces the frequency of coughing; the VitaloJAK cough monitor has been most frequently used [21]. The clinically meaningful threshold for cough frequency is thought to be a 30% reduction from baseline [22]. Secondary endpoints are generally patient-reported outcomes utilised to confirm the reductions in cough frequency observed are sufficient to be clinically meaningful to patients. As there is some debate about the methods used to develop these tools and their validity, a range of patient-reported outcomes are employed, typically including cough-specific quality of life (e.g. the Leicester Cough Questionnaire), cough severity visual analogue scales, cough severity diaries, and also the patient global impression of change [23–25].

## Current Standard Care for Refractory Chronic Cough

As already described, in many countries there is still no licenced therapy for RCC, and in those where licensing has been achieved, there are still issues to be resolved around reimbursement and access for patients. Consequently, those caring for patients with more severe RCC have little choice but to resort to prescribing unlicenced therapies. The use of these is typically based on small single-centre drug repurposing studies, the results of which often lack replication. Nonetheless, in the absence of other options, treatments such as gabapentin and low-dose slow-release morphine sulphate are recommended in national and international guidelines [3, 26].

### Low-Dose Morphine Sulphate

The first double-blind randomised controlled trial to report positive findings of any drug in refractory chronic cough utilised 5 and 10 mg of slow-release morphine sulphate given twice daily for a month compared to placebo [27]. This small, single-centre, crossover trial reported positive effects on cough-specific quality of life measured by the Leicester Cough Questionnaire (LCQ) and also on a cough severity numerical score. The main side effect was constipation, occurring in 40% of patients, but the treatment was otherwise well tolerated. Clinical experience of low-dose morphine suggests approximately 60% of RCC patients trialled with this therapy in a specialist clinic obtained a good response [28]. A further single-centre double-blind randomised crossover trial selecting patients reporting clinical benefit with low-dose morphine, found cough frequency to be reduced by over 70% after just five days of therapy, suggesting it can be highly effective in responders [29]. The main concern about use of low-dose morphine has been potential for addiction and abuse. It has therefore been recommended for the treatment of RCC in the European Respiratory Society cough guidance, but not in countries where opioid abuse is a more significant concern, such as the USA [3, 26].

A multi-centre double-blind randomised crossover trial in patients with cough associated with idiopathic pulmonary fibrosis also recently reported positive findings. MST slow-release 5mg provided a 40% reduction in cough frequency over placebo accompanied by improvements in patient-reported outcomes [30, 31]. Morphine is primarily an agonist at the mu opioid receptor and is assumed to act on inhibitory pathways in the brain. Why such low doses, compared with those used for analgesia, are so effective in some patients with chronic cough is unknown.

### Gabapentinoids

Gabapentin is a calcium channel modulator originally developed for the treatment of seizures but latterly licenced as a therapy for neuropathic pain. It has action in both the central and peripheral nervous system and therefore tends to be associated with significant CNS side effects, including sedation and unsteadiness. It also has abuse potential and is therefore a controlled drug. Following uncontrolled open-label studies suggesting benefit in patients with chronic cough [32, 33], a single-centre double-blind randomised controlled trial reported improvements cough-specific quality of life (LCQ) over placebo [34]. Although cough frequency monitoring was performed, this was just for one hour during the study visit and following capsaicin challenge, therefore those results are likely to be unreliable. Furthermore, gabapentin has positive effects on both mood and anxiety, potentially confounding patient-reported outcomes. No large-scale multi-centre double-blind randomised controlled trials have been performed.

### Speech and Language Therapy

Speech and language therapy techniques were first described as improving chronic cough in a single-centre randomised controlled trial compared with healthy lifestyle advice in 87 patients with RCC [35]. Although an unvalidated PRO was used, the intervention appeared to have impacts on cough, voice, throat symptoms, and symptom limitation after 4 therapy sessions over a 2-month period. A similar sized study recruiting at 3 hospital sites investigated a similar intervention delivered by speech and language therapists and physiotherapists compared with a sham therapy [36]. The primary endpoint for this study was the LCQ which improved by 1.5 points over the sham. Cough frequency improved by approximately 40% more than in the sham-treated arm at 4 weeks and seemed to be maintained at 3 months. No larger-scale trials have been completed.

Speech and language therapy is a complex intervention, comprising components of education, cough suppression techniques, vocal hygiene, and psychoeducational counselling. Thus, it is difficult to standardise the intervention and currently, it is not clear whether all or just some of the components are essential for efficacy. In practice, the therapy seems to be most effective when delivered by experienced therapists, but these are not widely available. There is also a question about the durability of the effects over longer timescales when patients may not continue to practise the techniques.

### Other Interventions

Amitriptyline is sometimes used to treat RCC based on a small double-blinded randomised trial in patients attending an otolaryngology service with post-viral chronic cough thought to result from a vagal neuropathy [37]. Amitriptyline 10 mg at night was compared with codeine 10 mg/guaifenesin 100 mg combined in a syrup (5 mls) taken every 6 h; no placebo arm was included in the study and the treatments were not matched. The majority of the patients treated with amitriptyline reported a 75–100% improvement in their cough, whereas most reported no improvement with codeine/guaifenesin.

The experience of superior laryngeal nerve block by the injection of local anaesthetic agents and corticosteroids has been described retrospectively following implementation in several clinics [38–40]. Recently, a small single-blind placebo-controlled study was performed comparing this treatment in 10 patients injected with active treatment and 7 with placebo, finding improvements in cough VAS and LCQ scores. Transient sensations of globus and soreness at the site of inject were the main adverse effects. Laryngeal botulin toxin injections have also been reported to produce improvements in series of patients in clinical care, but no controlled studies have been performed [41]. The broad safety of these interventions and duration of any effect currently remains unclear.

## Novel Therapies in Development

### P2X3 Antagonism

The first novel therapy found to have significant effects in patients with RCC was gefapixant, a first in class P2X3 antagonist [42]. P2X3 receptors are ion channels found on sensory afferent nerve fibres, activated by adenosine triphosphate (ATP). In pre-clinical studies, vagal C fibres, including those thought to be important in mediating cough have been shown to express P2X3 and P2X2 [43]. At present it is unclear whether ATP concentrations are elevated or P2X3 receptor expression increased in the airways of patients with RCC or how antagonism of P2X3 plays a role in reducing coughing to a range of chemical irritants, temperature changes, and mechanical stimuli. Nonetheless, in clinical trials P2X3 receptor antagonism has provided robust reductions in cough frequency and patient-reported outcomes.

#### Gefapixant

Originally planned to be developed as an analgesic, gefapixant (also previously known as AF-219 and MK-7624) has become the first therapeutic to undergo systematic development as a treatment for RCC, following unprecedented reductions in cough frequency seen in a small single-centre double-blind crossover trial; 75% reduction over placebo [42]. Initial studies utilised the maximum tolerated doses of 300–600 mg bd; however, it emerged that this treatment level produced ageusia, loss of taste, in all participants. This adverse effect reversed on discontinuation and is thought to result from the modest selectivity of gefapixant for P2X3 channels over heteromeric P2X2/3 channels found on the nerve fibres innervating the taste buds [44]. Fortunately, dose ranging studies suggested that antitussive effects were retained at much lower doses 30–50 mg bd, where taste was altered rather than lost and hence the therapy was better tolerated [45]. Larger multi-centre parallel group studies were performed in the UK and the USA [46] followed by the first ever global phase three trials of an antitussive treatment for RCC, which reported positive findings over placebo for a 45 mg bd dose (reformulated, equivalent to previous 50 mg dose) [20].

Several challenges in evaluating antitussive treatments emerged during the development of gefapixant. Whilst small placebo effects were observed in the initial crossover phase 2 studies, subsequent parallel group studies exhibited progressively larger improvements in the placebo arms, i.e. 37% to 57% reductions in cough frequency accompanied by comparable reductions in patient-reported outcomes [20, 46]. The apparent pharmacological effect of gefapixant 45/50mg remained remarkably consistent with reductions in cough frequency of 58 to 63% across phase 2 and 3 studies, but the effect over placebo was diminished by the increasing placebo responses. Also in the phase 3 trials, 60–70% of patients taking gefapixant 45 mg experienced taste disturbances, the majority of which were mild to moderate in intensity. Finally, although taste side effects were reported in all study arms, these were most frequent in 45-mg gefapixant-treated patients, risking unmasking of patients to their treatment allocation influencing the results.

Of note, a study of gefapixant in patients with chronic cough associated with IPF did not find such robust effects of this treatment [47]. There were some issues with the study conduct including failure of the randomisation process for some patients and the planned statistical analysis was not optimal. Nonetheless, post hoc analysis of the data and the responder analysis suggested a subgroup of patients may have benefitted from the treatment. In contrast, a study in healthy volunteers with acute cough associated with experimental rhinovirus infection found no effect of gefapixant compared with placebo [48].

#### Eliapixant and Filapixant

Following the taste side effects reported for gefapixant, more selective P2X3 antagonists were evaluated for the treatment of RCC; however, there was some uncertainty about whether effects at both P2X3 and P2X2/3 channels were both contributing to antitussive efficacy and hence whether more selective agents would have similar efficacy. Eliapixant and filapixant both demonstrated efficacy in dose ranging studies but eliapixant appeared to cause less taste disturbance (up to 21% of patients) and was therefore progressed to a phase 2b parallel trial [49, 50]. Although this trial reported positive findings, a small number of cases of liver toxicity prevented further development of this therapy for RCC [51].

#### Sivopixant

Another more selective P2X3 antagonist, sivopixant, exhibited promising findings in a single-dose crossover study, very similar in design to the first gefapixant study [52]. The reduction in daytime cough frequency of 32% over placebo (primary endpoint) was not quite statistically significant but taste adverse effects were only reported in 6.4% of patients. In a follow-up multi-centre parallel group study assessing a range of doses for 4 weeks, no dose of sivopixant could be discriminated from the very large placebo effect; 60% placebo reduction in cough frequency from baseline [53]. The largest absolute change in cough frequency was observed for the highest 300 mg dose but 30% of patients reported taste adverse effects. No further studies of sivopixant in RCC have been planned.

#### Camlipixant

Finally, thought to be the most selective P2X3 antagonist, camlipixant is the second compound in this class to be evaluated in phase 3 trials, ongoing at the time of writing [54]. The first double-blind randomised controlled crossover trial of camlipixant studied escalating doses from 25 mg to 200 mg versus matched placebo. Although the primary endpoint of awake cough frequency did not reach statistical significance, preplanned subgroup analysis in patients with a cough frequency ≥20 coughs per hour (80% of patients) and those with greater than the median cough frequency (≥32 coughs per hour, 50% of patients) exhibited significant improvements versus placebo for all doses tested [55]. This preplanned analysis was based on observations from several of the gefapixant studies that suggested P2X3 antagonism was most efficacious in patients with the highest baseline cough frequency [42, 46].

In a follow-up phase 2b parallel group study, several changes were made to the study design [56]. First, to address the increasing placebo effects seen in trials of other therapies, a single-blind 16-day placebo run-in period was implemented. Second, based on the phase 2a study results, only patients with ≥25 coughs per hour we recruited to the main study population, although an exploratory population examined a small number of patients with lower cough frequencies. These adjustments paid off in the study results which showed a smaller placebo effect than in previous parallel group studies (21%) and reductions of 34.4% and 34.2% in 24 cough frequency over placebo for the 50 and 200 mg doses, respectively [57]. In keeping with the selectivity of the compound taste adverse effects occurred in ≤6.5% of patients for all doses. Camlipixant is currently being evaluated in two large-scale phase 3 studies, again in patients selected for higher cough frequencies. The SME who developed the compound to this stage has recently been bought out by GlaxoSmithKline for $2 billion.

### Other Mechanisms of Action Under Investigation

The studies completed to date investigating P2X3 antagonists have typically found that between a quarter and a third of patients do not experience the 30% reduction in cough frequency thought to be the meaningful clinical threshold, suggesting some heterogeneity in the mechanisms underlying RCC. Furthermore, patients with less frequent/severe coughing than those recruited to these trials may not benefit from treatments interrupting the ATP–P2X3 axis. Therefore, treatments with alternative modes of action are required to optimally manage RCC patients.

#### Sodium Channel Blockade

Topical lidocaine has long been used to control coughing during bronchoscopy and is also sometimes utilised in clinical practice to treat RCC [58]. Lidocaine blocks the voltage-gated sodium channels important in the initiation of action potentials and their conduction. When objectively assessed in a small randomised controlled trial it has been shown to reduce coughing by about 50% for an hour after being sprayed on the oropharynx [59]. This was more effective than nebulisation, probably because nebulisation into the lower airways has an irritant effect and evokes coughing initially. So, the antitussive effects of topical lidocaine are relatively short lived and also associated with numbness in the mouth and lips preventing patients from safely eating after treatment. Efforts have therefore been made to develop similar therapies with a longer duration of action and without loss of sensation.

A novel approach to sodium channel blockade has been developed using a compound that is only active in blocking sodium channels after entering neurones via large pore ion channels, such as P2X3 channels [60]. A phase 2a clinical trial has been performed but the results are not yet published.

#### TRPM8 Agonism

Activation of TRPM8 ion channels produces cooling sensations. One new therapy has used an orally dissolving tablet containing a TRPM8 agonists (AX-8) placed on the back of the tongue to act as a counter irritant to the sensations of throat irritation reported by many patients with RCC [16]. In a double-blind randomised controlled trial, AX-8 reduced cough frequency but not significantly over 8 h, the duration of action suggested by a previous open-label study [61]. However, the effect was significant over 4 h and exaggerated in those patients reporting greater throat discomfort, consistent with the proposed mechanism of action. Further studies in this subgroup of patients are hoped to confirm efficacy.

#### Opioid Receptor Agonism

Nalbuphine is an agonist at kappa opioid receptors and an antagonist at mu opioid receptors, where morphine is an agonist. It is therefore perhaps surprising that it has been shown to be an effective antitussive agent in a small double-blind crossover trial in patients with cough associated with IPF [62]. In a double-blind randomised controlled trial, extended release nalbuphine tablets reduced cough frequency by 50.8% over placebo. This was accompanied by improvements in PROs and therefore, this is the first therapy to show robust effects on chronic cough in IPF. Further larger-scale studies and IPF and RCC are awaited.

#### NK-1 Antagonism

Following a positive study testing aprepitant as a cough treatment in patients with lung cancer, there has been interest in the potential antitussive effects of centrally acting neurokinin-1 (NK-1) antagonists [63]. Following a negative trial in RCC, a double-blind randomised controlled trial is currently in progress testing the effects of orvepitant in patients with cough associated with IPF [64, 65].

### What Interventions Haven’t Worked?

Prior to the first study assessing gefapixant, no new therapy had been found to be efficacious in RCC, although a number of therapies had been assessed using validated endpoints. Some of these studies have suggested certain targets are not relevant to the treatment of RCC; however, in others, limitations of the therapies, trial design, or dosing mean the targets may still be important.

#### Peripherally Acting Therapies

TRPV1 antagonists have been expected to be efficacious based on the large body of evidence showing heightened cough responses to the TRPV1 ligand capsaicin in RCC. However, two different oral TRPV1 antagonists assessed in small double-blind randomised controlled trials had no impact on spontaneous cough frequency, despite evidence of target engagement, in the form of reduced cough responses to capsaicin [66, 67]. This suggests this particular pathway is redundant in RCC patients. A further study in patients with cough and COPD found similar results [68].

A single very small study has assessed an oral TRPV4 antagonist utilising an adaptive study design employing futility analysis to seek evidence for efficacy [69]. Pre-clinical models have suggested that non-neuronal TRPV4 receptors are capable of releasing ATP and therefore indirectly activating P2X3 channels [70]. A double-blind randomised controlled crossover trial comparing drug to matched placebo for seven days was performed. Futility analysis on complete data from 15 patients showed no evidence of an antitussive effect. It should be noted however that target engagement was not assessed in the study and dosing was limited by toxicity and therefore the target should not be ruled out.

Finally, several inhaled drugs have also been tested to treat cough, but none have reported positive findings. There are several challenges with the inhaled route for treating cough. If the treatment is even a weak irritant, it may evoke coughing during administration in chronic cough patients. This is a problem not only for tolerability but also efficacy as the treatment is rapidly ejected by coughing. In addition, it is unclear exactly what the optimal airway deposition pattern might be for an inhaled antitussive therapy. Two pan-sodium channel blockers have been found to evoke rather than reduce coughing in studies in RCC and IPF patients [71, 72]. A double-blind randomised controlled study has also been performed using an inhaled TRPA1 antagonist [73]. The study results were not published but are believed to be negative. Lastly, inhaled cromoglycate, which has an unclear mechanism of action, did not have any antitussive effect in a multi-centre trial in patients with IPF and chronic cough [74].

#### Centrally Acting Therapies

Several studies, not all fully published, have tested NMDA receptor antagonists in RCC. The over-the-counter cough medicine dextromethorphan is an antagonist at this receptor, although the evidence for its antitussive efficacy is weak. Furthermore, NMDA receptors are implicated in the central nervous system processes mediating central sensitisation which may be relevant to RCC [75, 76]. In an open-label dose-escalating study of oral memantine, RCC patients struggled to escalate the dose due to adverse effects; however, there was some suggestion of efficacy with a reduction in cough frequency of 25% from baseline at the maximum tolerated dose [77]. A couple of placebo-controlled studies were also performed using a novel formulation of memantine aiming to improve tolerability in acute and chronic cough, however, the results of these were not published [78, 79]. Another open-label study of a novel NMDA receptor antagonist with MAOI activity was also poorly tolerated in RCC patients [80]. More recently, an open-label study of ifenprodil has been performed in IPF patients with chronic cough finding a 39.4% reduction from baseline at 12 weeks; the study is not yet fully published and placebo-controlled studies are awaited [81].

GABA_B_ agonists have long been thought to have antitussive effects either resulting from their impact on transient lower oesophageal sphincter relaxations, reducing gastro-oesophageal reflux or directly through an inhibitory effect on the neuronal pathways mediating cough. Several uncontrolled studies have suggested baclofen, a centrally acting GABA_B_ agonist, reduced cough responses to inhaled capsaicin [82, 83]. However central nervous system side effects are problematic and include seizures if withdrawn suddenly. More recently, a peripherally acting GABA_B_ agonist, lesogaberan, has been studied. Interestingly unlike baclofen, this therapy did not reduce capsaicin evoked cough in healthy volunteers [84]. However, capsaicin responses were significantly improved in a double-blind randomised controlled crossover trial in RCC patients despite a 25% reduction in cough frequency over placebo that did not reach the statistical significance [85]. The efficacy seemed to be unrelated to baseline measures of reflux or the temporal associations between cough and reflux events. These findings would seem to suggest more of an effect on the neuronal pathways mediating cough rather than an effect on gastro-oesophageal reflux. Dosing in this study was limited by concerns about hepatic toxicity and so it remains unknown whether a higher dose of this treatment may have been more efficacious.

Other central targets that have not been able to demonstrate efficacy as antitussive agents include several NK-1 antagonists and a nicotinic agonist. Following an open-label study, orvepitant was evaluated in a parallel group phase 2b study in RCC but cough frequency did not reduce significantly over the reductions seem in the placebo arm [65, 86]. Interestingly, there were positive changes in some of the PROs but as NK-1 antagonists are anxiolytic, these findings are difficult to interpret. A second centrally acting NK-1 antagonist, serlopitant similarly reported negative findings [87]. Bradanicline, a nicotinic acetylcholine receptor agonist, was assessed in a double-blind randomised crossover trial but neither escalating doses of bradanicline nor placebo-reduced cough frequency [88].

## Conclusion

**Fig. 3:**
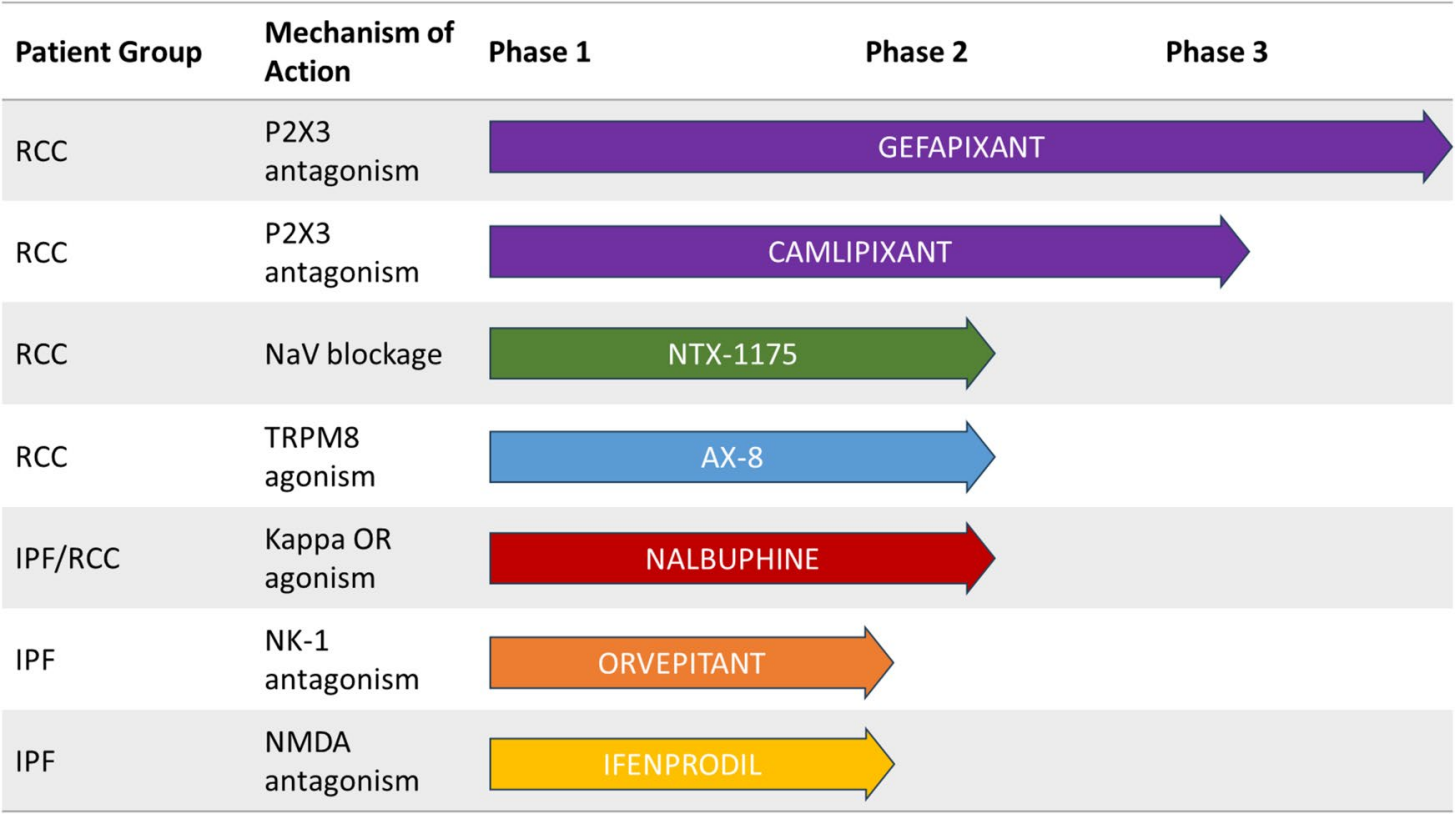
Current antitussive pipeline 2023. *RCC* refractory chronic cough, *IPF* idiopathic pulmonary fibrosis, *NaV* voltage-gated sodium channel, *TRPM8* transient receptor vanilloid potential melastatin 8, *OR* opioid receptor, *NK-1* neurokinin-1, *NMDA* N-methyl-d-aspartate

As can be appreciated from this review, an expanding range of both central and peripheral neuronal targets have been explored the treatment of chronic cough with several progressing in clinical development, see Fig. 3. These have yielded evidence for some of the first effective antitussive agents with P2X3 antagonists for RCC. Opioid receptor agonists also show promise for the treatment of chronic cough associated with IPF. However, marked placebo effects have become evident in some of these trials, affecting even objective measures of cough frequency and these are proving a challenge to exceed with pharmacological effects. A better understanding of the processes contributing to the effects seen in placebo-treated patients and how to mitigate them is going to be essential future studies.

## Data Availability

No datasets were generated or analysed during the current study.
